# Association between initial 30‐day cumulative clozapine dose and risk of agranulocytosis: A nationwide register‐based cohort study

**DOI:** 10.1111/pcn.70092

**Published:** 2026-06-15

**Authors:** Yuki Kikuchi, Xue Li, Hiroshi Komatsu, Norio Yasui‐Furukori, Ken Inada, Hiroaki Tomita

**Affiliations:** ^1^ Department of Psychiatry Graduate School of Medicine, Tohoku University Sendai Japan; ^2^ Department of Psychiatry Kodama Hospital Ishinomaki Japan; ^3^ The Japanese Society of Clinical Neuropsychopharmacology Tokyo Japan; ^4^ Department of Regional Alliance for Promoting Liaison Psychiatry Tohoku University Graduate School of Medicine Sendai Japan; ^5^ Department of Psychiatry Tohoku University Hospital Sendai Japan; ^6^ Department of Psychiatry School of Medicine, Dokkyo Medical University Mibu Japan; ^7^ Department of Psychiatry Kitasato University School of Medicine Sagamihara Kanagawa Japan

**Keywords:** CPMS, inflammation, myocarditis, neutropenia, personalized titration

## Abstract

**Aims:**

Clozapine‐induced agranulocytosis (CIA) is traditionally considered an idiosyncratic, dose‐independent reaction. However, emerging evidence suggests that clozapine‐related inflammatory events may exhibit dose‐dependent characteristics, leading us to hypothesize that CIA risk may similarly relate to early cumulative exposure.

**Methods:**

Using Japan's nationwide Clozaril Patient Monitoring Service database (2009–2024), the 30‐day cumulative dose following initiation was calculated for all treated patients. Participants were stratified into quartiles (Q1–Q4). CIA was defined as two consecutive absolute neutrophil counts <500/mm^3^. The incidence of CIA was compared across quartiles using Kaplan–Meier survival curves and Cox proportional hazards models adjusted for age and sex.

**Results:**

Among 18,189 patients (mean age 42.9 [SD 12.7]; 46.4% female), 138 (0.76%) developed CIA, with no cases occurring within the first 30 days. The incidence rates of CIA in Q1, Q2, Q3, and Q4 were 0.44%, 0.66%, 0.74%, and 1.20%, respectively. Compared with Q1, hazard ratios (HRs) over 180 days were 1.78 (95% confidence interval [CI] 0.96–3.30; *P* = 0.0659), 2.01 (95% CI 1.10–3.66; *P* = 0.0233), and 3.07 (95% CI 1.74–5.42; *P* < 0.001) for Q2, Q3, and Q4, respectively. Male sex and age >40 years were identified as independent risk factors. *E*‐values for Q4 HR were 5.59 (point estimate) and 2.88 (95% CI), indicating high robustness against unmeasured confounders such as co‐medications.

**Conclusions:**

Higher early cumulative clozapine exposure was associated with increased CIA risk. These findings suggest that early exposure may serve as a clinically relevant marker of CIA risk, although causal inference is limited and further research is needed.

Clozapine is the most effective antipsychotic for treatment‐resistant schizophrenia (TRS); however, it is widely underused due to serious adverse effects, particularly hematological complications. In the 1970s, clozapine was withdrawn from the market due to reports of fatal agranulocytosis. However, in 1988, Kane *et al*. demonstrated its efficacy against TRS,^1^ and it was reintroduced with strict blood monitoring.^2^ Although blood monitoring has significantly reduced the rate of mortality due to clozapine‐induced agranulocytosis (CIA),^3^ it has substantially impeded the widespread use of clozapine.

There is increasing evidence suggesting that overly strict blood monitoring overestimates the CIA risk relative to the benefits of clozapine in reducing mortality in patients with TRS.^4^, ^5^, ^6^ Accordingly, the US Food and Drug Administration discontinued the Clozapine Risk Evaluation and Mitigation Strategies system in June 2025.^7^ Recent international consensus guidelines recommend weekly absolute neutrophil count (ANC) monitoring for 18 weeks after clozapine initiation, followed by monitoring at 4‐week intervals and discontinuation of routine ANC monitoring after 2 years.^8^ However, weekly blood monitoring for 18–26 weeks after clozapine initiation remains mandatory in many countries. Beyond early detection of CIA through strict blood monitoring, there is currently no established method to prevent its onset.

The pathophysiological mechanism underlying CIA remains unclear. However, specific human leukocyte antigen (HLA) haplotypes have been implicated in CIA,^9^ suggesting that immune‐related processes may play a role.^10^ In addition, reactive metabolites generated during clozapine bioactivation have been proposed as potential contributors to toxicity.^11^ However, clinical studies have generally not demonstrated a clear association between clozapine dosage and CIA risk,^12^ and CIA has therefore been regarded as largely dose‐independent. At the same time, growing evidence indicates that rapid clozapine titration is associated with an increased risk of inflammatory adverse events.^13^, ^14^, ^15^, ^16^, ^17^, ^18^ These observations raise the possibility that early cumulative exposure, rather than dose at a single time point, may be relevant to CIA risk. We therefore hypothesized that early cumulative clozapine exposure during the initial titration phase may be associated with subsequent CIA risk. The aim of this study was to examine the association between CIA risk and early cumulative clozapine exposure during the initial titration phase in a nationwide cohort.

## Methods

### The Clozaril Patient Monitoring Service in Japan

In Japan, clozapine is only indicated for TRS. Only Clozaril Patient Monitoring Service (CPMS)‐certified psychiatrists can register patients in the CPMS and initiate clozapine treatment in an inpatient setting at CPMS‐certified hospitals. TRS is diagnosed when treatment with two antipsychotics (chlorpromazine equivalent ≥600 mg/day) for 4 weeks is ineffective, or when two antipsychotics cannot be administered in sufficient doses due to extrapyramidal symptoms. White blood cell count (WBC) and ANC monitoring is performed weekly for 26 weeks after clozapine initiation, biweekly for the next 26 weeks, and quadweekly thereafter. Clozapine should be discontinued if the WBC or ANC is <3,000/mm^3^ or <1,500/mm^3^, respectively, followed by daily blood tests until the WBC and ANC are ≥4,000/mm^3^ and ≥2,000/mm^3^, respectively. After that, weekly blood tests should be performed for 4 weeks. Additionally, the 4‐week follow‐up blood test is mandatory not only for neutropenia but also for clozapine discontinuation for any reason. In the CPMS database, WBC and neutrophil percentage are recorded, and ANC is automatically calculated; direct entry of ANC is also permitted. The ANC values recorded in the database were used in this study.

### Ethical procedures

All procedures contributing to this work complied with the ethical standards of the relevant national and institutional committees on human experimentation and with the Declaration of Helsinki of 1975, as revised in 2008. This study was approved by the Ethics Committee of the Tohoku University Graduate School of Medicine (Approval ID: 2023‐1‐856/857), which waived the requirement for informed consent given the anonymity of the CPMS data.

### Study design and participants

We used comprehensive data from the CPMS database, encompassing all records from its inception on 29 July 2009, through 31 December 2024 (21,962 patients and 1,787,040 blood tests). Among them, we included 18,189 patients after excluding those who were rechallenged with clozapine and those who were reregistered due to hospital transfer. Apart from 22 patients who had personally imported clozapine and 48 patients with prior participation in clinical trials before CPMS enrolment,^19^ all patients were receiving clozapine for the first time. We collected the following data from the CPMS database: age at clozapine initiation, sex, blood test date, WBC, ANC, date of clozapine initiation, clozapine dosage on the blood test date, and number of days prescribed. No information regarding the ethnicity of participants was included. CIA was defined as two consecutive ANCs <500/mm^3^ (Taylor's criteria for true agranulocytosis).^20^, ^21^ Patients were divided into quartiles (Q1–Q4) based on their 30‐day cumulative clozapine dose following initiation. The 30‐day exposure window was selected primarily to ensure temporal separation between exposure assessment and CIA onset, as no CIA cases occurred within the first 30 days after clozapine initiation in this cohort. In addition, this period approximately corresponded to the initial titration phase in routine clinical practice.

### Statistical analysis

The primary exposure was the 30‐day cumulative clozapine dose, calculated for each patient using a step‐function method (last observation carried forward), assuming the prescribed dose remained constant until the next prescription record.

The primary endpoint was time to CIA onset, defined as the interval from clozapine initiation to the first occurrence of CIA or censoring at the last follow‐up. Censored events included clozapine discontinuation for reasons other than CIA, continued clozapine treatment until 31 December 2024 (end of data collection period), and transfer to another hospital. Kaplan–Meier curves were used to estimate survival for each quartile, with between‐curve differences assessed using the log‐rank test. The hazard ratio (HR) and 95% confidence intervals (CIs) were calculated for each quartile over 180 days after clozapine initiation, with Q1 as the reference. We performed five sensitivity analyses on the Kaplan–Meier estimates. (i) We performed fixed‐horizon logistic regression analyses for time windows other than the 180‐day primary analysis period (60, 90, 120, and 150 days). (ii) We conducted a sensitivity analysis excluding patients who discontinued or temporarily withheld clozapine within 30 days of treatment initiation. (iii) We performed a sensitivity analysis using moderate neutropenia (ANC < 1000/mm^3^ on two consecutive occasions) as the outcome measure, which has a less stringent criterion than the primary analysis. (iv) To evaluate the robustness of the exposure definition, additional sensitivity analyses were conducted using alternative exposure windows of 14 and 60 days. For the 14‐day analysis, patients were stratified into tertiles of cumulative clozapine dose. Because the 14‐day cumulative dose had many tied values due to standardized early titration schedules, quartile classification did not yield approximately equal‐sized groups and resulted in substantial imbalance. Therefore, for the 14‐day sensitivity analysis, we categorized patients into tertiles to obtain more balanced and interpretable exposure groups. For the 60‐day analysis, patients were stratified into quartiles, and a landmark approach was used, with follow‐up beginning at day 60 for patients who were event‐free and remained at risk at that time. (v) As an additional sensitivity analysis, we examined the relationship between titration speed and the CIA risk. Titration speed was calculated as the difference between the clozapine dose at week 5 (i.e. 4 weeks after initiation) and the standard starting dose of 12.5 mg (as specified in the Japanese package insert) divided by 4 weeks to approximate the average weekly dose increase during the initial titration phase. Patients were stratified into quartiles based on titration speed, and Kaplan–Meier and Cox proportional hazards analyses were performed.

We assessed the association between cumulative clozapine dose and CIA risk using multivariable Cox proportional‐hazards regression models, with HRs and 95% CIs as effect estimates. Cumulative dose quartile was included as a categorical variable in a single Cox proportional hazards model, with Q1 as the reference group. Models were adjusted for sex and age group (<30, 30–39, 40–49, and ≥50 years), with <30 years as the reference group. Schoenfeld residuals verified the proportional‐hazards assumption. To assess the robustness of the observed association to potential unmeasured confounding, we calculated *E*‐values for the estimated hazard ratios. The *E*‐value represents the minimum strength of association that an unmeasured confounder would need to have with both the exposure and the outcome, on the risk ratio scale, to fully explain the observed association, conditional on the measured covariates. *E*‐values were calculated based on the point estimates and the lower bounds of the 95% CIs. The incidence rate of CIA was calculated per 1000 person‐years with 95% Poisson CIs. All statistical analyses were two‐sided, with statistical significance set at *P* < 0.05.

## Results

### Characteristics of participants and the incidence of CIA in the total cohort

This study included 18,189 patients (mean age at clozapine initiation: 42.9 [standard deviation 12.7]; females, 46.4%; Table 1). During the entire study period, 138 (0.76%) patients developed CIA. The median duration (interquartile range) until CIA onset was 78 (58–112) days. The shortest and longest durations until onset were 38 and 1259 days, respectively. Among the 138 CIA cases, 63.0% (87/138), 89.8% (124/138), 96.3% (133/138), and 98.5% (136/138) occurred within 90, 180, 364, and 728 days, respectively. The incidence rate of CIA per 1,000 person‐years was 16.0 within 180 days, 1.48 during 181–364 days, and 0.12 after 364 days (Table S1).

**Table 1:** Demographic characteristics of the participants

	All	Q1	Q2	Q3	Q4
Number of participants	18,189	4,583	4,513	4,584	4,509
Female, *n* (%)	8,448 (46.4%)	2,183 (47.6%)	2,157 (47.8%)	2,068 (45.1%)	2,040 (45.2%)
Age, mean (SD)	42.9 (12.7)	44.3 (13.5)	43.4 (12.8)	42.4 (12.2)	41.4 (12.1)
Age, year, *n* (%)					
<20	487 (2.7%)	133 (2.9%)	111 (2.5%)	125 (2.7%)	118 (2.6%)
20–29	2,657 (14.6%)	630 (13.7%)	612 (13.6%)	679 (14.8%)	736 (16.3%)
30–39	4,563 (25.1%)	1,013 (22.1%)	1,112 (24.6%)	1,170 (25.5%)	1,268 (28.1%)
40–49	5,113 (28.1%)	1,222 (26.7%)	1,296 (28.7%)	1,326 (28.9%)	1,269 (28.1%)
50–59	3,599 (19.8%)	973 (21.2%)	909 (20.1%)	919 (20.0%)	798 (17.7%)
>60	1,770 (9.7%)	612 (13.4%)	473 (10.5%)	365 (8.0%)	320 (7.1%)
Initial white blood cell count, median (IQR)	5900 (5100–7030)	5750 (4900–6895)	5900 (5050–7020)	6000 (5150–7100)	6000 (5180–7200)
Initial absolute neutrophil count, median (IQR)	3398 (2757–4312)	3307 (2680–4208)	3390 (2748–4292)	3444 (2785–4382)	3458 (2819–4378)
Events of CIA, *n* (%)	138 (0.76%)	20 (0.44%)	30 (0.66%)	34 (0.74%)	54 (1.20%)
Time to event, day, median (min, IQR, max)	78 (38, 58–112, 1259)	97 (47, 72–163, 1133)	82 (40, 63–120, 503)	76 (39, 57–97, 1259)	72 (38, 56–108, 544)
Time to event, day, *n* (%)					
≤90	87 (63.0%)	8 (40.0%)	19 (63.3%)	22 (64.7%)	38 (70.4%)
90–180	37 (26.8%)	8 (40.0%)	9 (30.0%)	10 (29.4%)	10 (18.5%)
180–364	9 (6.5%)	3 (15.0%)	1 (3.3%)	1 (2.9%)	4 (7.4%)
364–728	3 (2.2%)	0 (0.0%)	1 (3.3%)	0 (0.0%)	2 (3.7%)
>728	2 (1.4%)	1 (5.0%)	0 (0.0%)	1 (2.9%)	0 (0.0%)
Final clozapine dose before the onset of CIA, mg, median (IQR)	300 (200–400)	225 (125–350)	250 (175–400)	300 (200–425)	350 (225–500)

CIA, clozapine‐induced agranulocytosis; IQR, interquartile range; max, maximum; min, minimum; SD, standard deviation.

### Association between 30‐day cumulative clozapine dose and risk of agranulocytosis

**Fig. 1:**
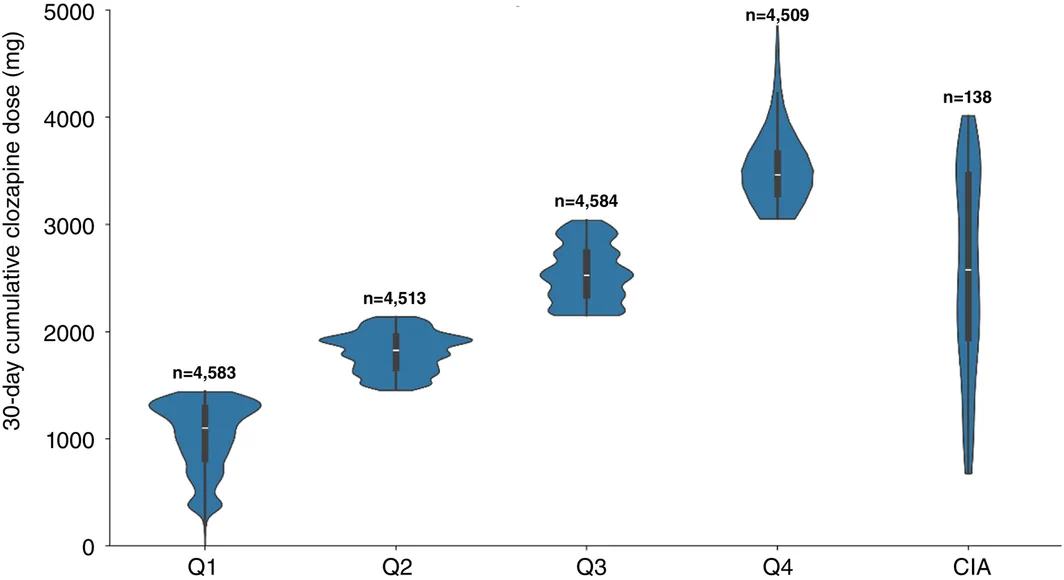
Distribution of 30‐day cumulative clozapine dose across quartiles (Q1–Q4) and patients with clozapine‐induced agranulocytosis (CIA).

Figure 1 and Table S2 show the distribution of the 30‐day cumulative clozapine doses across quartiles (Q1–Q4) and patients who developed CIA. There was a balanced distribution of participants across the quartiles, with ≈4,500 patients in each group. Baseline WBC and ANC were comparable across the quartiles. The median 30‐day cumulative dose increased stepwise from Q1 (1,100 mg) to Q4 (3,463 mg). Patients with CIA had a broad cumulative dose distribution that overlapped with the higher quartiles, with a median 30‐day cumulative dose of 2,575 mg. The incidence rates of CIA in Q1, Q2, Q3, and Q4 were 0.44% (20/4583), 0.66% (30/4513), 0.74% (34/4584), and 1.20% (54/4509), respectively (Table 1). The incidence of CIA increased across quartiles of 30‐day cumulative clozapine dose. Furthermore, the median duration until CIA onset was 97 days in Q1, 82 days in Q2, 76 days in Q3, and 72 days in Q4 (Table 1). The median time to CIA onset was shorter in higher cumulative‐dose quartiles. Moreover, the median clozapine dose at CIA onset increased across cumulative‐dose quartiles (225 mg/day in Q1, 250 mg/day in Q2, 300 mg/day in Q3, and 350 mg/day in Q4).

### Kaplan–Meier survival curves

**Fig. 2:**
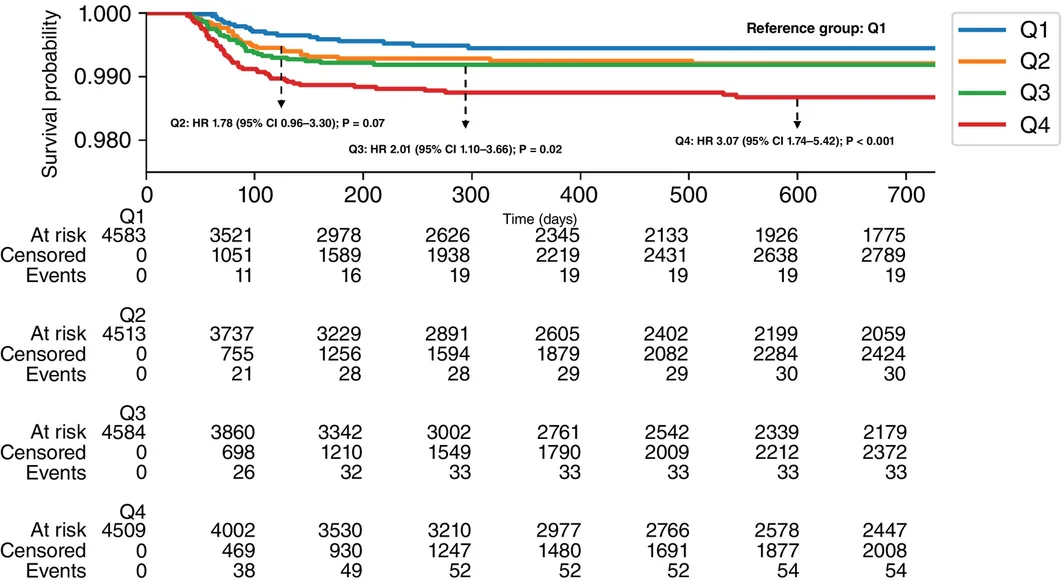
Kaplan–Meier survival curves stratified by quartiles of 30‐day cumulative clozapine dose.

Figure 2 shows the Kaplan–Meier survival curves for the incidence of CIA stratified by quartiles. There was a clear dose–response relationship, with higher quartiles exhibiting steeper declines in survival probability compared with the lowest quartile (Q1).

Over the 180‐day analysis period, the HRs in Q2, Q3, and Q4 compared to Q1 were 1.78 (95% CI 0.96–3.30; *P* = 0.0659), 2.01 (95% CI 1.10–3.66; *P* = 0.0233), and 3.07 (95% CI 1.74–5.42; *P* < 0.001), respectively. Table S3 shows sensitivity analyses of Kaplan–Meier models across different fixed horizons (60, 90, 120, 150, and 180 days). The risk estimates for Q3 and Q4, compared with Q1, remained consistently elevated for all analysis periods. A sensitivity analysis excluding patients who discontinued or temporarily stopped clozapine within the first 30 days yielded results consistent with the primary analysis, showing a higher risk of CIA in Q4 than in Q1 (HR 2.72 [95% CI 1.62–4.56]; *P* = 0.000144) (Fig. S1). Furthermore, in a sensitivity analysis with the outcome changed to moderate neutropenia (ANC < 1000/mm^3^ on two consecutive occasions), although the difference in HRs decreased, Q4 still showed a significantly higher risk of CIA compared with Q1 (HR 1.98 [95% CI 1.38–2.83]; *P* = 0.000183) (Fig. S2). Additional analyses using alternative exposure windows (14 and 60 days) yielded similar patterns of association (Figs. S3 and S4, Table S4). In an additional analysis stratified by initial titration speed, higher‐titration‐speed groups were also associated with an increased risk of CIA (Fig. S5, Table S5). The distribution of cumulative dose and titration speed across exposure groups is presented in Table S6.

### Multivariable Cox regression analysis

Multivariable Cox regression analysis demonstrated that a higher 30‐day cumulative clozapine dose was associated with an increased risk of CIA (Table 2). Compared with Q1, the HR was 1.49 (95% CI: 0.84–2.62; *P* = 0.17) for Q2, 1.67 (95% CI: 0.96–2.91; *P* = 0.068) for Q3, and 2.73 (95% CI: 1.63–4.56; *P* < 0.001) for Q4. Compared with male sex, female sex was associated with a significantly lower risk of CIA (HR: 0.66, 95% CI: 0.47–0.94; *P* = 0.020). Older age was strongly associated with increased risk, particularly in the 40–49 (HR 3.02, 95% CI: 1.47–6.20; *P* = 0.0026) and ≥50 (HR 5.50, 95% CI: 2.75–11.0; *P* < 0.001) years age groups compared with the <30 years age group.

**Table 2:** Multivariable Cox proportional hazards regression for CIA

Covariate	HR	LCI	UCI	*P*
Q2 (second quartile of 30‐day cumulative dose, reference = Q1)	1.486	0.844	2.617	0.170
Q3 (third quartile of 30‐day cumulative dose, reference = Q1)	1.672	0.962	2.907	0.068
Q4 (fourth quartile of 30‐day cumulative dose, reference = Q1)	2.727	1.631	4.561	0.00013
Female (vs male)	0.662	0.468	0.937	0.020
Age 30–39 years (reference <30 years)	1.049	0.454	2.424	0.911
Age 40–49 years	3.022	1.473	6.201	0.0026
Age ≥50 years	5.497	2.747	11.000	0.0000015

CIA, clozapine‐induced agranulocytosis; HR, hazard ratio; LCI, lower confidence interval; UCI, upper confidence interval.

## Discussion

### Association between CIA risk and early clozapine exposure

Clozapine‐induced inflammatory adverse events, such as myocarditis and fever, are increasingly recognized as dose‐dependent phenomena.^13^, ^14^, ^15^, ^16^, ^17^, ^18^ In contrast, the CIA has traditionally been considered an idiosyncratic reaction independent of drug concentration. In this nationwide cohort study, we observed a dose–response relationship between early cumulative clozapine exposure and CIA risk during the initial titration phase, with the highest‐dose group (Q4) exhibiting a three‐fold greater risk of CIA compared to the lowest‐dose group (Q1). In addition, the time to CIA onset tended to be shorter in the higher‐dose quartile groups. Similar patterns were observed in sensitivity analyses excluding patients who discontinued or temporarily withheld clozapine within the first 30 days. Analyses using alternative exposure windows (14 and 60 days) yielded generally comparable associations. Analyses stratified by initial titration speed also showed higher CIA risk in the higher‐exposure groups. Because titration speed and cumulative dose are closely related during the initial treatment phase, this analysis should be interpreted as an alternative parameterization of early exposure intensity rather than an independent validation of the main findings. These findings support the relevance of early exposure intensity, although the underlying mechanisms remain unclear.

Previous studies have reported no clear association between clozapine dose and agranulocytosis risk. For example, Alvir *et al*. did not find a significant relationship using a matched case–control design.^12^ However, their analysis focused on the maximal dose, the last recorded dose, and the cumulative dose up to the index date rather than on early cumulative exposure during a predefined titration window. In addition, the relatively small number of cases may have limited statistical power to detect dose–response patterns. Therefore, their findings do not directly address the role of early exposure in CIA risk.

The present findings suggest that early cumulative exposure may be relevant to CIA risk, although the underlying biological mechanisms remain incompletely understood. While dose‐dependent toxicity related to clozapine bioactivation has been proposed,^11^, ^22^, ^23^, ^24^ these observations should not be interpreted as evidence of a shared mechanism with other inflammatory adverse events.

In clinical practice, dose escalation is influenced not only by tolerability but also by the severity of psychotic symptoms and the need for rapid symptom control. Therefore, the observed association between higher early cumulative dose and CIA risk may partly reflect treatment decisions driven by clinical status (confounding by indication). Evidence linking titration strategies to psychiatric symptom trajectories remains limited, highlighting the need to balance efficacy and safety when interpreting these findings.

### Temporal pattern of CIA risk following clozapine initiation

In the present study, the risk of CIA declined markedly over time, with the incidence rate falling from 16.0 per 1,000 person‐years within the first 180 days to 1.48 during the 181–364‐day period, and further to 0.12 after 1 year. The period of elevated risk observed between weeks 4 and 18, followed by a sharp decline, is consistent with previous registry studies.^4^, ^5^

Clozapine‐associated inflammatory reactions, such as myocarditis, typically occur within the first few weeks after treatment initiation, whereas CIA generally develops later, often after the first month. This temporal difference suggests that these adverse reactions are unlikely to represent a single continuous process. Although both may involve immune‐related mechanisms and may be influenced by early drug exposure, the biological pathways underlying CIA remain incompletely understood.

The present findings are consistent with the possibility that early clozapine exposure may be associated with later CIA onset after a latency period. These findings suggest that CIA risk is concentrated during the early treatment period, although the relative contributions of early exposure, subsequent dose escalation, and proximal exposure remain unclear.

Current international consensus guidelines recommend intensive hematological monitoring during the initial treatment phase, followed by reduced frequency after approximately 18 weeks.^8^ The very low incidence observed after 1 year in our cohort is consistent with this approach. These findings may support consideration of similar adjustments to monitoring strategies in Japan, while taking into account local clinical and regulatory contexts.

### Association of sex and age with CIA risk

In the present study, male sex was associated with an increased risk of CIA. However, previous findings regarding sex differences in CIA risk have been inconsistent. Studies by Northwood *et al*. and Alvir *et al*. have suggested a higher risk of CIA in females.^4^, ^12^ However, in the study by Alvir *et al*., the association was attenuated and no longer statistically significant after adjustment for age. In addition, sex was not a primary focus of the study by Northwood *et al*., and the reported effect sizes were modest. Taken together, these findings indicate that evidence for sex differences in CIA risk remains limited and inconsistent.^25^

Regarding age, we observed a significant increase in CIA risk among patients aged ≥40 years, with a particularly marked increase in those aged ≥50 years. This finding is consistent with several previous studies. For example, analysis of a large clozapine registry demonstrated an approximately 50% increase in agranulocytosis risk per decade of age.^26^ Similarly, a nationwide Japanese cohort based on earlier CPMS data reported that patients with agranulocytosis were significantly older than those without hematological events.^19^ The present findings, based on an expanded and more recent dataset, are broadly consistent with earlier observations from the same nationwide registry. In contrast, a meta‐analysis by Myles *et al*. found that categorical age groups were associated with differences in CIA risk, with higher incidence observed in older populations; however, mean age was not significantly associated with risk in meta‐regression analyses, suggesting that the association may depend on the analytical approach or population characteristics.^3^ Taken together, these findings suggest that age‐related vulnerability to CIA is likely present but may not be uniform across all populations or analytical frameworks. One possible explanation is that older individuals may have reduced hematopoietic reserve or increased susceptibility to drug‐induced bone marrow suppression. However, these mechanisms remain speculative and require further investigation.

### Clinical implications

This study demonstrates a dose–response relationship rather than identifying a specific clinical threshold. Given the substantial inter‐individual variability in clozapine metabolism, affected by factors such as smoking status, body weight, and genetic background, it would be premature to define a universal safe titration rate based on these observational data.

In Japan, the package‐insert titration schedule recommends reaching 200 mg/day over 21 days (≈66 mg/week), corresponding to a 30‐day cumulative dose of approximately 3,937.5 mg. This value lies near the upper end of the observed distribution in our cohort, whereas most patients in routine clinical practice received slower titration. For context, the lowest quartile (Q1) – which was associated with the lowest CIA incidence – corresponds to an approximate titration rate of 12.5 mg/week (30‐day cumulative dose ≈975–1,100 mg). These observations suggest that clinicians in Japan may already be adopting more cautious approaches than those described in the package insert; however, they should not be interpreted as evidence that the package‐insert schedule itself increases CIA risk.

International recommendations also support slower titration, particularly in East Asian populations.^17^, ^18^ For example, guidance by de Leon *et al*. corresponds to 30‐day cumulative doses of approximately 3,000 mg for normal metabolizers and 1,700 mg for poor metabolizers,^17^ broadly aligning with the middle quartiles observed in this study. Together, these findings are consistent with the value of individualized titration while emphasizing the need to balance safety with timely symptom improvement.

Very slow titration may delay clinical response or prolong hospitalization, and evidence regarding this trade‐off remains limited. However, moderate titration strategies have been reported to achieve symptom improvement while maintaining tolerability.^27^ Overall, the present findings support a cautious and individualized approach to early dose escalation rather than a specific numeric target. Further studies are needed to determine titration strategies that minimize CIA risk while preserving the therapeutic benefits of clozapine.

### Future research

Our findings suggest that early cumulative clozapine exposure is associated with CIA risk, highlighting the potential importance of interindividual differences in clozapine metabolism. Because clozapine metabolism is influenced by patient‐specific factors, such as smoking status, inflammation, and obesity,^28^ future studies should clarify how these factors contribute to variability in CIA susceptibility.

In addition, genetic factors may play an important role. For example, the HLA‐B*59:01 allele has been associated with CIA risk in Japanese patients,^29^ raising the possibility that genotype‐informed risk stratification could improve safety. Other factors, including concomitant valproic acid use^30^ and differences in metabolic capacity among populations (such as low metabolism in East Asians),^12^, ^17^, ^18^ also warrant systematic investigation.

Although evidence specific to CIA remains limited, international recommendations for clozapine‐induced myocarditis have adopted individualized titration strategies based on patient characteristics.^17^, ^18^ These approaches provide a conceptual framework for tailoring early clozapine exposure, but their applicability to CIA risk remains to be established. Accordingly, further research is needed to clarify how titration strategies influence CIA risk.

Prospective studies are needed to evaluate personalized titration strategies, such as early monitoring of clozapine plasma levels to estimate metabolic capacity and guide dose escalation.^31^ Such approaches may help identify patients at higher risk and enable safer, individualized dosing.

Finally, further research is needed to better characterize the biological processes linking early exposure to CIA onset.^31^, ^32^ Integrating genetic, metabolic, and clinical factors may ultimately support the development of precision titration strategies that minimize CIA risk while preserving the therapeutic benefits of clozapine.

### Limitations and strengths

This study has several limitations. First, due to the retrospective design and the nature of the CPMS database, adjustment was limited to age and sex. Other potential confounders – including disease severity, concomitant medications, smoking status, body weight, comorbidities, and genetic background – were not available in this dataset. Smoking induces CYP1A2 and may lead to higher prescribed doses, while body weight and genetic susceptibility, such as HLA‐B*59:01, could influence individual sensitivity to clozapine. Illness severity may influence early dosing decisions, as clinicians may escalate doses more rapidly in severely ill patients. Although no clear biological mechanism links psychiatric severity to CIA, residual confounding by indication cannot be excluded. Concomitant medications associated with both initial clozapine dosage and CIA risk – such as valproic acid – may act as confounders.

To assess the potential impact of unmeasured confounding, we calculated *E*‐values. The observed hazard ratio of 3.07 (Q4 vs Q1) corresponded to an *E*‐value of 5.59 (2.88 for the lower confidence bound), indicating that a confounder with a large effect size would be required to fully explain the observed association. While residual confounding cannot be excluded, these findings suggest that it would be unlikely to entirely account for the dose–response relationship. For context, concurrent use of valproate has been associated with clozapine‐associated neutropenia, with an adjusted odds ratio of 2.28 in a large register‐based case–control study,^33^ which is substantially smaller than our calculated *E*‐value. However, the potential influence of multiple interacting confounders warrants further investigation in prospective studies.

Second, our analysis focused on the 30‐day cumulative dose as a baseline exposure marker. However, dosing patterns and the rate of escalation after the first 30 days, particularly immediately before the onset of CIA, may also influence risk. Because early cumulative dose may correlate with subsequent dosing patterns, it is difficult to distinguish whether the observed association reflects early exposure alone or sustained higher exposure later in the course. Our study did not explicitly model these subsequent dosing dynamics as time‐dependent variables. Therefore, the 30‐day cumulative dose should be interpreted as a proxy for early titration intensity rather than the sole determinant of CIA risk. Future studies incorporating time‐dependent exposures would help clarify these relationships.

Third, the cohort was predominantly Japanese, and generalizability to other populations may be limited. Fourth, we could not follow patients transferred to other hospitals. However, only 2.3% of the cohort was transferred within 180 days, and no CIA cases were recorded prior to transfer, suggesting that the impact of loss to follow‐up was negligible (Table S7).

Despite these limitations, this study has several strengths. It utilizes a large, nationwide database encompassing all patients treated with clozapine in Japan, enabling reliable evaluation of a rare outcome. In addition, the temporal sequence between early exposure and CIA onset was clearly established, and a consistent dose–response pattern was observed. These findings provide new insight into a reaction traditionally considered idiosyncratic.

### Conclusion

In conclusion, this nationwide cohort study found that higher initial cumulative clozapine exposure was associated with an increased risk of CIA during the early phase of treatment. The risk was concentrated within the first several months after initiation. These findings support the relevance of early cumulative exposure as a clinically meaningful marker of CIA risk. However, because of the observational design, these results should not be interpreted as evidence of a causal relationship or a specific underlying mechanism. Further research is needed to clarify the biological pathways and to determine optimal titration strategies that balance safety and efficacy.

## Author contributions

Y.K., H.K., and H.T. conceived of the study. Y.K., X.L., H.K., N.Y.‐F., K.I., and H.T. contributed to the methodology and data acquisition. Y.K. and X.L. analyzed the data. Y.K. and X.L. had access to the data and verified the data. Y.K. and X.L. wrote the first draft of the manuscript. Y.K. and X.L. contributed equally to this work as co–first authors. All authors were responsible for the interpretation of the results and the decision to submit the manuscript.

## Disclosure statement

Y.K. received speaker fees from Janssen Pharmaceutical K.K.; Meiji Seika Pharma Co., Ltd.; Sumitomo Pharma Co., Ltd.; and Takeda Pharmaceutical Company Limited. X.L. declares no conflicts of interest. H.K. received lecture fees from Meiji Seika Pharma Co., Ltd.; Sumitomo Pharma Co., Ltd.; Takeda Pharmaceutical Company Limited; and Boehringer Ingelheim. N.Y.‐F. has received speaker fees from Daiichi Sankyo, EA Pharma, Eisai, Janssen, Kyowa Kirin, Hisamitsu, Lundbeck, Meiji Seika Pharma, Mitsubishi Tanabe Pharma, Eli Lilly, Mochida, MSD, Otsuka, Shionogi, Sumitomo Pharma, Takeda, Tsumura, Viatris, and Yoshitomi Yakuhin; and consulting fees from Mochida, Boehringer Ingelheim, Bristol Myers Squibb, and EA Pharma. K.I. has received personal fees from the following companies over the past 3 years: EA Pharma, Viatris Pharmaceuticals Japan, Eisai, MSD, Otsuka Pharmaceutical, Ono Pharmaceutical, Kyowa Kirin, Kowa, Shionogi, Sumitomo Pharma, Daiichi Sankyo Company, Takeda Pharmaceutical, Mitsubishi Tanabe Pharma, Nipro, Nippon Chemiphar, Eli Lilly Japan, Boehringer Ingelheim Japan, Nexera Pharma, Novartis Pharma, Nobelpharma, Pfizer Japan, Meiji Seika Pharma, Mochida Pharmaceutical, Janssen Pharmaceutical, and Lundbeck Japan. H.T. received research funding, directed to Tohoku University, from Daiichi Sankyo Company, Limited; Eisai Co., Ltd.; Otsuka Pharmaceutical Co., Ltd.; and Sumitomo Dainippon Pharma Co., Ltd. H.T. also received personal honoraria from Daiichi Sankyo Company, Limited; EA Pharma Co., Ltd.; Eisai Co., Ltd.; Janssen Pharmaceutical K.K.; Lundbeck; Meiji Seika Pharma Co., Ltd.; Mochida Pharmaceutical Co., Ltd.; MSD K.K.; Mylan EPD G.K.; Otsuka Pharmaceutical Co., Ltd.; Pfizer Japan Inc.; Sumitomo Pharma Co., Ltd.; Takeda Pharmaceutical Company Limited; and Viatris Inc. This study has not received any funding.

## Supporting information


**Figure S1.** Kaplan–Meier curves by 30‐day cumulative clozapine dose (excluding discontinuation).

**Figure S2.** Kaplan–Meier curves by 30‐day cumulative clozapine dose for two consecutive neutrophil counts <1000/μL.

**Figure S3.** Kaplan–Meier survival curves for clozapine‐induced agranulocytosis (CIA) stratified by tertiles of 14‐day cumulative.

**Figure S4.** Kaplan–Meier survival curves for clozapine‐induced agranulocytosis (CIA) stratified by quartiles of 60‐day cumulative.

**Figure S5.** Kaplan–Meier survival curves for clozapine‐induced agranulocytosis (CIA) stratified by quartiles of initial titration speed.

**Table S1.** Incidence rate of agranulocytosis per 1000 person‐years.
**Table S2.** Distribution of 30‐day cumulative clozapine dose across quartiles (Q1–Q4) and agranulocytosis (CIA) cases.
**Table S3.** Sensitivity analysis of Kaplan–Meier analysis across different follow‐up horizons.
**Table S4.** Cox proportional hazards regression analyses for sensitivity analyses using alternative exposure windows (14‐day cumulative dose tertiles and 60‐day cumulative dose quartiles with a landmark analysis at day 60).
**Table S5.** Cox proportional hazards regression analyses for sensitivity analyses stratified by initial titration speed.
**Table S6.** Distribution of cumulative clozapine dose and initial titration speed across exposure groups used in sensitivity analyses.
**Table S7.** Transfer‐related censoring analysis by cumulative dose quartiles (Q1–Q4).

## Data Availability

The CPMS data is available upon request to Novartis Pharma through appropriate procedures.
