# Supplementary material for: Association between initial 30‐day cumulative clozapine dose and risk of agranulocytosis: A nationwide register‐based cohort study
Source: Psychiatry Clin Neurosci. 2026 Jun 15;80(9):765–72. doi: 10.1111/pcn.70092 (PMC13537344; doi:10.1111/pcn.70092)
Supplement: Supplementary file 1 [file PCN-80-765-s001.docx]

| **Table S1. Incidence rate of agranulocytosis per 1000 person-years** | | | | |  |  |
| --- | --- | --- | --- | --- | --- | --- |
| **Phase** | **Group** | **Events (n)** | **Person-years (PY)** | **Incidence rate (per 1000 PY)** | **LCI (95% CI)** | **UCI (95% CI)** |
| ≤180 days | All | 124 | 7742.03 | 16.02 | 13.32 | 19.10 |
|  | Q1 | 16 | 1834.75 | 8.72 | 4.98 | 14.16 |
|  | Q2 | 28 | 1919.53 | 14.59 | 9.69 | 21.08 |
|  | Q3 | 32 | 1970.20 | 16.24 | 11.11 | 22.93 |
|  | Q4 | 48 | 2017.56 | 23.79 | 17.54 | 31.54 |
| 181–364 days | All | 9 | 6092.09 | 1.48 | 0.68 | 2.80 |
|  | Q1 | 3 | 1371.82 | 2.19 | 0.45 | 6.39 |
|  | Q2 | 1 | 1497.81 | 0.67 | 0.017 | 3.72 |
|  | Q3 | 1 | 1561.29 | 0.64 | 0.016 | 3.57 |
|  | Q4 | 4 | 1661.18 | 2.41 | 0.66 | 6.17 |
| ≥364 days | All | 5 | 42251.47 | 0.12 | 0.038 | 0.28 |
|  | Q1 | 1 | 8094.30 | 0.12 | 0.0031 | 0.69 |
|  | Q2 | 1 | 9728.87 | 0.10 | 0.0026 | 0.57 |
|  | Q3 | 1 | 11107.15 | 0.090 | 0.0023 | 0.50 |
|  | Q4 | 2 | 13321.15 | 0.15 | 0.018 | 0.54 |
|  |  |  |  |  |  |  |
| Column definitions: | |  |  |  |  |  |
| Phase: Number of days after clozapine initiation. | | |  |  |  |  |
| Group: Subgroup classification (e.g., Q1–Q4 of 30-day cumulative clozapine dose). | | | | |  |  |
| Events (n): Number of agranulocytosis cases observed. | | | |  |  |  |
| Person-years (PY): Total follow-up duration for the group, expressed as person-years. | | | | |  |  |
| Incidence rate (per 1000 PY): Calculated as the number of events divided by person-years, multiplied by 1000. | | | | | |  |
| LCI (95% CI): Lower 95% confidence interval bound for the incidence rate. | | | |  |  |  |
| UCI (95% CI): Upper bound of the 95% confidence interval for the incidence rate. | | | | |  |  |

| **Table S2. Distribution of 30-day cumulative clozapine dose across quartiles (Q1–Q4) and agranulocytosis (CIA) cases** | | | | | | |
| --- | --- | --- | --- | --- | --- | --- |
| **Group** | **Minimum (mg)** | **First Quartile (Q1, mg)** | **Median (mg)** | **Third Quartile (Q3, mg)** | **Maximum (mg)** | **N (samples)** |
| Q1 | 0 | 812.5 | 1100 | 1287.5 | 1437.5 | 4583 |
| Q2 | 1450 | 1662.5 | 1825 | 1950 | 2137.5 | 4513 |
| Q3 | 2150 | 2337.5 | 2525 | 2737.5 | 3037.5 | 4584 |
| Q4 | 3050 | 3287.5 | 3462.5 | 3662.5 | 18000 | 4509 |
| CIA | 675 | 1940.625 | 2575 | 3462.5 | 4012.5 | 138 |
|  |  |  |  |  |  |  |
| Column definitions: | |  |  |  |  |  |
| Group: Stratification group based on quartiles of 30-day cumulative clozapine dose (Q1–Q4) or agranulocytosis (CIA) cases. | | | | | | |
| Minimum / Maximum (mg): The lowest and highest 30-day cumulative dose observed in each group. | | | | | | |
| First Quartile (Q1) / Third Quartile (Q3) (mg): The 25^th^ and 75^th^ percentiles of the distribution. | | | |  |  |  |
| Median (mg): The 50^th^ percentile of the distribution. | | |  |  |  |  |
| N (samples): Total number of samples included in each group. | | |  |  |  |  |

| **Table S3. Sensitivity analysis of Kaplan–Meier analysis across different follow-up horizons** | | | | | |
| --- | --- | --- | --- | --- | --- |
| **Horizon (days)** | **Group** | **Odds ratio (OR)** | **95% CI (Lower)** | **95% CI (Upper)** | **P-value** |
| 60 | Q2 | 7.118 | 0.875 | 57.878 | 0.066 |
| 60 | Q3 | 11.022 | 1.422 | 85.403 | 0.022 |
| 60 | Q4 | 18.365 | 2.451 | 137.624 | 0.0046 |
| 90 | Q2 | 2.418 | 1.057 | 5.529 | 0.036 |
| 90 | Q3 | 2.758 | 1.227 | 6.201 | 0.014 |
| 90 | Q4 | 4.860 | 2.265 | 10.430 | 0.000049 |
| 120 | Q2 | 1.866 | 0.922 | 3.775 | 0.083 |
| 120 | Q3 | 2.425 | 1.236 | 4.758 | 0.010 |
| 120 | Q4 | 3.754 | 1.980 | 7.116 | 0.000051 |
| 150 | Q2 | 2.116 | 1.090 | 4.106 | 0.027 |
| 150 | Q3 | 2.394 | 1.251 | 4.580 | 0.0084 |
| 150 | Q4 | 3.783 | 2.047 | 6.991 | 0.000022 |
| 180 | Q2 | 1.782 | 0.963 | 3.298 | 0.066 |
| 180 | Q3 | 2.007 | 1.100 | 3.662 | 0.023 |
| 180 | Q4 | 3.071 | 1.742 | 5.416 | 0.00011 |
|  |  |  |  |  |  |
| Horizon (days): Fixed time window (in days) used for survival analysis (60–180 days). | | | | | |
| Group: Quartile group of 30-day cumulative dose (Q1–Q4), with Q1 as the reference. | | | | | |
| OR (Odds ratio): Odds ratio of agranulocytosis occurrence compared with Q1 (reference group). | | | | | |
| 95% CI (Lower / Upper): Lower and upper bounds of the 95% CI for OR. | | | | | |
| P-value: Statistical significance of the comparison. | | | |  |  |

**Table S4. Cox proportional hazards regression analyses for sensitivity analyses using alternative exposure windows (14-day cumulative dose tertiles and 60-day cumulative dose quartiles with a landmark analysis at day 60)**

| **14-day cumulative dose** | | | |
| --- | --- | --- | --- |
| **Variable** | **HR** | **95% CI** | **P value** |
| **T2 vs T1** | 1.40 | 0.91–2.16 | 0.126 |
| **T3 vs T1** | 1.63 | 1.06–2.49 | 0.026 |
| **Female (vs Male)** | 0.65 | 0.46–0.92 | 0.015 |
| **Age (Years)** | 1.06 | 1.04–1.07 | <0.001 |
| **60-day cumulative dose (landmark analysis)** | | | |
| **Q2 vs Q1** | 1.14 | 0.60–2.17 | 0.70 |
| **Q3 vs Q1** | 1.80 | 1.00–3.27 | 0.04 |
| **Q4 vs Q1** | 2.37 | 1.33–4.20 | 0.003 |
| **Female (vs Male)** | 0.46 | 0.30–0.70 | <0.001 |
| **Age (Years)** | 1.06 | 1.04–1.07 | <0.001 |

Values are hazard ratios (HRs) with 95% confidence intervals (CIs) derived from Cox proportional hazards models adjusted for age and sex.

Age was included as a continuous variable.

For the 14-day analysis, patients were stratified into tertiles (T1–T3) of cumulative clozapine dose, with T1 representing the lowest exposure group.

For the 60-day analysis, patients were stratified into quartiles (Q1–Q4) of cumulative clozapine dose, with Q1 representing the lowest exposure group.

A landmark approach was applied to the 60-day analysis, including only patients who remained at risk at day 60, with follow-up beginning at that time.

**Table S5. Cox proportional hazards regression analyses for sensitivity analyses stratified by initial titration speed**

| **Variable** | **HR** | **95% CI** | **P value** |
| --- | --- | --- | --- |
| **Q2 vs Q1** | 1.33 | 0.79–2.24 | 0.283 |
| **Q3 vs Q1** | 2.03 | 1.31–3.16 | 0.002 |
| **Q4 vs Q1** | 2.93 | 1.81–4.76 | <0.001 |
| **Female (vs Male)** | 0.64 | 0.46–0.91 | 0.013 |
| **Age (Years)** | 1.06 | 1.05–1.07 | <0.001 |

Values are hazard ratios (HRs) with 95% confidence intervals (CIs) derived from Cox proportional hazards models adjusted for age and sex.

Age was included as a continuous variable.

Q1–Q4 indicate quartiles of initial titration speed, with Q1 representing the slowest titration group.

**Table S6. Distribution of cumulative clozapine dose and initial titration speed across exposure groups used in sensitivity analyses**

| **14-day cumulative dose tertiles** | | | | |
| --- | --- | --- | --- | --- |
| **Group** | **N** | **Events** | **Dose range (mg)** | **Median (IQR)** |
| **T1** | 6063 | 35 | 0–287.8 | 212.5 (175–262.5) |
| **T2** | 6255 | 50 | 300–550 | 437.5 (362.5–437.5) |
| **T3** | 5871 | 53 | 562.5–8400 | 612.5 (612.5–662.5) |
| **60-day cumulative dose quartiles (landmark analysis)** | | | | |
| **Group** | **N** | **Events** | **Dose range (mg)** | **Median (IQR)** |
| **Q1** | 4137 | 18 | 284.4–5312.5 | 3875 (2762.5–4662.5) |
| **Q2** | 4148 | 19 | 5325–7800 | 6500 (5850–7112.5) |
| **Q3** | 4115 | 28 | 7812.5–10525 | 9150 (8462.5–9662.5) |
| **Q4** | 4133 | 36 | 10537.5–36000 | 12450 (11362.5–14050) |
| **Initial titration speed quartiles** | | | | |
| **Group** | **N** | **Events** | **Speed range (mg/week)** | **Median (IQR)** |
| **Q1** | 7085 | 35 | -3.125–21.875 | 15.625 (9.375–21.875) |
| **Q2** | 3792 | 24 | 25–34.375 | 34.375 (28.125–34.375) |
| **Q3** | 4940 | 47 | 37.5–46.875 | 46.875 (46.875–46.875) |
| **Q4** | 2372 | 32 | 50–146.875 | 59.375 (59.375–71.875) |

Values represent the distribution of cumulative clozapine dose or titration speed within each exposure group.

For the 14-day analysis, patients were stratified into tertiles (T1–T3) of cumulative clozapine dose.

For the 60-day analysis, patients were stratified into quartiles (Q1–Q4) of cumulative clozapine dose, and a landmark approach was applied, including only patients who remained at risk at day 60, with follow-up beginning at day 60.

The initial titration speed was calculated as the difference between the clozapine dose at week 5 and the starting dose of 12.5 mg, divided by 4 weeks.

| **Table S7. Transfer-related censoring analysis by cumulative dose quartiles (Q1–Q4)** | | | | | |
| --- | --- | --- | --- | --- | --- |
| **Row** | **All** | **Q1** | **Q2** | **Q3** | **Q4** |
| Events of transfer, n (%) | 1306 (7.2%) | 240 (5.2%) | 268 (5.9%) | 391 (8.5%) | 407 (9.0%) |
| Time to transfer, day, median (min, IQR, max) | 329 (10, 142–962, 5452) | 279 (14, 131–812, 3915) | 302 (10, 144–804, 4319) | 333 (14, 150–899, 5054) | 406 (21, 147–1215, 5452) |
| Time to transfer, day, n (%) |  |  |  |  |  |
| ≤90 | 161 (12.3%) | 31 (12.9%) | 35 (13.1%) | 48 (12.3%) | 47 (11.5%) |
| 90–180 | 260 (19.9%) | 56 (23.3%) | 51 (19.0%) | 75 (19.2%) | 78 (19.2%) |
| 180–364 | 260 (19.9%) | 46 (19.2%) | 65 (24.3%) | 82 (21.0%) | 67 (16.5%) |
| 364–728 | 206 (15.8%) | 41 (17.1%) | 40 (14.9%) | 66 (16.9%) | 59 (14.5%) |
| ≥728 | 419 (32.1%) | 66 (27.5%) | 77 (28.7%) | 120 (30.7%) | 156 (38.3%) |
|  |  |  |  |  |  |
| Q1–Q4: Quartiles of 30-day cumulative clozapine dose (Q1 = lowest quartile, Q4 = highest quartile). | | | |  |  |
| Events of transfer, n (%): Number (n) and percentage (%) of patients censored due to hospital transfer. | | | |  |  |
| Time to transfer, day, median (min, IQR, max): Distribution of time (days) from clozapine initiation to hospital transfer, presented as median, minimum, interquartile range (IQR), and maximum. | | | | | |
| Time to transfer, day, n (%): Number (n) and percentage (%) of patients transferred within each time window: ≤90, 90–180, 180–364, 364–728, and ≥728 days. | | | | | |
